# A Small-Molecule Compound Targeting Canine Mammary Cancer Regulates CXCL10 and MECOM Transcripts via Histone Modifications in CMT-N7

**DOI:** 10.3390/ani15152274

**Published:** 2025-08-04

**Authors:** Rongrong Wang, Chuyang Zhu, Xiaoyue Yuan, Cuipeng Zhu, Saber Y. Adam, Haoyu Liu, Demin Cai, Jiaguo Liu

**Affiliations:** 1 MOE Joint International Research Laboratory of Animal Health and Food Safety and Institute of Traditional Chinese Veterinary Medicine, College of Veterinary Medicine, Nanjing Agricultural University, Nanjing 210095, China; njaurong@163.com; 2 Alovet Co., Ltd., Suzhou 215021, China; 3 Jiangsu Key Laboratory of Animal Genetic Breeding and Molecular Design, College of Animal Science and Technology, Yangzhou University, Yangzhou 225009, China; zhuchuyang@foxmail.com (C.Z.); yu120447@outlook.com (X.Y.); cpzhu0318@outlook.com (C.Z.); saaber5757@gmail.com (S.Y.A.); haoyu.liu@yzu.edu.cn (H.L.)

**Keywords:** RORγ, canine mammary cancer, inflammation, histone modifications

## Abstract

Nuclear receptors are involved in multiple biological processes, among which RORγ can regulate the expression of inflammation-related genes and is thus frequently used as a therapeutic target for cancer. Canine mammary cancer is one of the most common tumor diseases in dogs, with a relative incidence rate of 46.71% for CMT in China over the past five years, severely threatening the life and health of dogs. Therefore, the search for novel drugs targeting canine mammary cancer is of great significance. This study aims to investigate how the RORγ inhibitors W6134 and XY018 affect the expression of inflammatory genes through histone modifications in CMT-N7 cells. These results show that W6134 and XY018 can upregulate signaling pathways related to inflammation and apoptosis and influence the expression of associated genes. The close link between RORγ and inflammation-related genes further confirms that RORγ may serve as a therapeutic target for canine cancer. Additionally, ChIP-qPCR was used to detect the enrichment of histone markers such as P300, H3K27ac, H3K4me1, H3K9la, and H3K9bhb at the target loci of CXCL10 and MECOM genes. Collectively, our findings provide molecular evidence for the protective role of RORγ in canine mammary cancer, potentially by regulating inflammatory pathways via histone modifications, offering new insights for improving the cure rate and survival of affected dogs.

## Simple Summary

This study aims to explore the potential therapeutic effects of two novel drugs, W6134 and XY018, on canine mammary cancer cells, while providing new targets for the treatment of canine mammary cancer. This work focused on the molecular mechanisms by which these two drugs inhibit cancer cell growth, particularly through histone modifications to regulate the inflammatory genes *CXCL10* and *MECOM*. The finding revealed that treatment with W6134 and XY018 upregulates apoptosis, cellular inflammatory signaling pathways, and alters the expression of related genes, providing a new perspective for the treatment of canine mammary cancer. Overall, our study provides molecular evidence that W6134 and XY018 may inhibit canine cancer cells by modulating inflammatory pathways.

## 1. Introduction

Nuclear receptors are a class of ligand-activated transcription factors involved in various biological processes, including cell growth, differentiation, metabolism, and the maintenance of organismal homeostasis [1]. In recent years, the role of nuclear receptors in breast cancer has garnered increasing attention [2]. Therapeutic targets for metabolic and autoimmune diseases are found in the RAR-related orphan receptor gamma (RORγ) subfamily of the nuclear receptor superfamily of transcription factors, which also includes the related RORα and RORβ [3]. The limited number of identified ligands makes RORγ a poorly characterized nuclear receptor [4]. However, RORγ can regulate the expression of the pro-inflammatory cytokine IL-17. Activation of RORγ can induce various IL-17-mediated autoimmune diseases and is also frequently utilized in anticancer therapies [5]. RORγ inhibitors W6134 and XY018 significantly decrease its transcriptional activity, which affects the expression of related genes [6]. Previous studies have highlighted the diverse biological effects of RORγ inhibitors, particularly their impact on cancer cells [7]. Additionally, RORγ inhibitors can affect normal cellular metabolism and function by suppressing the expression of the NLRP3 inflammasome, further exacerbating inflammatory responses [8]. They also significantly inhibit cell proliferation and promote apoptosis [9]. Therefore, given the broad therapeutic benefits of RORγ inhibitors W6134 and XY018, the use of nuclear receptor inhibitors as a pharmacological intervention for cancer represents an emerging therapeutic target.

With the growing importance of canine health, increased attention to mammary gland health in dogs is essential [10]. Canine mammary tumors (CMTs), one of the most common neoplastic diseases in dogs [11], which are malignant in approximately 40–50% of cases [12]. Mammary cell cultures serve as a convenient tool for in vitro studies of mammary biology [13]. Key factors that affect the occurrence and development of CMTs include breed [14], age [15], genetic susceptibility [16], hormones [17], diet [18], and cyclooxygenase-2 (COX-2) expression [19]. These factors contribute to abnormal proliferation and differentiation of mammary epithelial cells, impairing normal cellular functions and structural integrity, ultimately leading to the development of CMTs, which pose a serious threat to canine health [20,21]. Therefore, inhibiting the growth of canine cancer cells is critical for preventing tumorigenesis and improving the cure and survival rates of affected dogs, highlighting the profound significance of research on CMTs.

However, as small molecular compounds, the effects of W6134 and XY018 on canine cancer cells from the perspective of metabolic regulation have not been thoroughly studied [21]. Transcriptome sequencing has made it feasible to map differentially expressed genes onto established pathways, thus clarifying the rationale behind experimental outcomes [22]. To deepen insights into how RORγ inhibitors act on canine cancer cells and define their functional traits, we acquired W6134 and XY018 from prior research and used transcriptomic analysis to evaluate their transcriptional regulatory impacts on such cells.

CXCL10 (C-X-C motif chemokine ligand 10), alternatively referred to as an interferon-inducible protein, is a chemokine mainly synthesized by diverse cell types (including monocytes, endothelial cells, fibroblasts, and activated T cells) as a response to interferon-γ (IFN-γ) and other pro-inflammatory cues [23]. Its primary function is to recruit activated T cells, NK cells, and monocytes expressing the CXCR3 receptor to sites of inflammation [24]. Studies have shown that low expression of CXCL10 is associated with cancer development [25]. MECOM is a multi-domain transcription factor [26] that plays a crucial role in regulating gene expression during early development and hematopoiesis [27]. Notably, studies have found that MECOM acts as a tumor suppressor gene, thus potentially serving as a target for the anticancer effects of W6134 and XY018 [28].

Epigenetic regulation plays a crucial role in gene expression [29]. Perturbations in these epigenetic pathways can induce histone modifications, including methylation, acetylation, phosphorylation, and ubiquitination [30], which are critical for governing gene expression and transcriptional processes. The promoter region is where DNA methylation usually takes place because it inhibits protein binding and, consequently, decreases gene transcription [31]. Additionally, epigenetic mechanisms can trigger inflammation and promote tumor development and transformation in inflammatory contexts [32]. Histone acetylation (P300, H3K27ac), a post-translational modification, modulates gene expression and correlates with cancer onset and progression [33]. In contrast to acetylation, histone 3 lysine 4 (H3K4) methylation is an evolutionarily conserved histone modification that significantly activates transcription [34]. Studies have shown that enhanced H3K4/H3K9 methylation affects global gene expression and eliminates breast cancer metastasis [35]. Gene transcription activation is related to H3K4 methylation, whereas transcriptional repression is linked to H3K9 methylation [36]. This suggests that histone modifications can promote transcription factor (TF) occupancy and transcriptional activation to influence the expression of *CXCL10* and *MECOM* [37]. Within this framework, RORγ inhibitors have been shown to impact histone modifications which are essential for governing gene expression across diverse pathological conditions. Although research on W6134 and XY018 in diverse breast cancers is growing, significant gaps remain in understanding their roles in canine mammary cancer cells and how they influence the key inflammatory gene expression such as *CXCL10* and *MECOM*. However, we hypothesize that RORγ inhibitors enhance the production of these transcripts through specific histone modifications, thereby affecting CMT-N7 cells. Therefore, this study aims to elucidate the mechanisms by which W6134 and XY018 regulate inflammatory pathway gene expression through histone modifications in CMT-N7 cells.

## 2. Materials and Methods

### 2.1. Cell Culture and Treatment

CMT-N7 cells [38] (Nanjing Agricultural University’s Veterinary Hospital, Nanjing, China) cultured in high-glucose DMEM medium (Cytiva, Shanghai, China) supplemented with 10% fetal bovine serum (Hyclone, Logan, UT, USA) and 1% penicillin-streptomycin (Solarbio, Beijing, China). The cells were kept in a humidified incubator under conditions of 37 °C and 5% CO_2_. The experiment was divided into three groups: (1) DMEM group (vehicle group), (2) W6134 (5 μM, MedChemExpress LLC, Monmouth Junction, NJ, USA) treatment for 48 h group, and (3) XY018 (5 μM, MedChemExpress LLC, Monmouth Junction, NJ, USA) treatment for 48 h group.

### 2.2. RNA Extraction and Sequencing

We achieved the extraction of total RNA by lysing the cells in the blank and treatment groups in 6-well plates with 1 mL of Trizol (Invitrogen, Waltham, MA, USA). Then, we evaluated the quality of RNA by using the Agilent Bioanalyzer 2100 system (Agilent Technologies, Santa Clara, CA, USA). We performed high-throughput sequencing by Wuhan Yingzi Gene Technology Co., Ltd., Wuhan, China, using the BGI T7 sequencing platform. The analysis of raw data was carried out using Xshell7 and Xftp7, and we selected the FPKM (Fragments Per Kilobase of exon per Million reads mapped) quantitative analysis method for subsequent data analysis.

### 2.3. Enrichment Analysis of Genes

We utilized the Gene Set Enrichment Analysis (GSEA 4.1.0) software to identify significantly enriched pathways. Additionally, by leveraging the Metascape database (available online: http://metascape.org/ (accessed on 3 January 2025)) and DAVID (available online: https://david.ncifcrf.gov/ (accessed on 15 January 2025)), statistical enrichment analysis and classification were conducted on the Gene Ontology (GO) and Kyoto Encyclopedia of Genes and Genomes (KEGG) pathways of the differentially expressed genes (DEGs). Data analysis and visualization were accomplished on the online platform (available online: http://www.bioinformatics.com.cn (accessed on 6 February 2025)), and GSEA enrichment analysis plots, KEGG enrichment Sankey diagrams, bubble plots, pathway diagrams, volcano plots, and bar charts of GO-pathway enrichment results were generated. Meanwhile, the STRING online functional protein interaction network platform (available online: https://cn.string-db.org/ (accessed on 7 February 2025)) was employed to perform correlation analysis on the differentially expressed genes.

### 2.4. Cell Counting and Viability Assay

Live cells were counted using a hemocytometer chamber under a microscope at 0, 24, and 48 h. In the experimental procedure, 96-well culture plates were used to seed cells, which were then subjected to treatment with W6134 (5 μM) or XY018 (5 μM) for 24 h and 48 h, respectively. Then, 10 μL of CCK8 solution was added to each well, and the plates were incubated in the dark for 3 h. After incubation, absorbance was measured at 450 nm using a microplate reader.

### 2.5. Calcein Staining

CMT-N7 cells were exposed to W6134 (5 μM) or XY018 (5 μM) for 48 h. After treatment, the medium was removed, and cells were washed twice with assay buffer. Subsequently, cells were incubated with calcein (1 μmol/L) at 37 °C for 30 min. Following incubation, cells were washed twice with phosphate-buffered saline (PBS), sealed with mounting medium, and observed under a fluorescence microscope.

### 2.6. CHIP-qPCR

CMT-N7 cells were treated through incubation with a 1% formaldehyde solution on a shaker for 12 min and then subjected to a 10-minute glycine incubation step. Following supernatant discard, cells underwent two PBS washes. Subsequently, 3 mL of PBS was dispensed into the dish, and a cell brush was used to scrape cells. The resulting suspension underwent centrifugation at 2000 rpm for 5 min at 4 °C. After the supernatant was discarded, cells were re-suspended in lysis buffer (1 mmol/L EDTA, 50 mmol/L HEPES pH 8.0, 0.5% NP-40, 140 mmol/L NaCl, 0.25% Triton X-100, 10% glycerol). A subsequent 2000 rpm centrifugation at 4 °C for 5 min was performed, with the supernatant discarded thereafter. Cells were then resuspended in wash buffer (1 mmol/L EDTA, 0.5 mmol/L EGTA, 10 mmol/L Tris pH 8.0, 200 mmol/L NaCl) and centrifuged again under identical conditions. After supernatant removal, resuspension in sonication buffer (0.1% SDS, 10 mmol/L Tris HCl pH 8.0, 1 mmol/L EDTA pH 8.0) were followed. Cells were sonicated, and the lysate was centrifuged at 12,000 rpm for 10 min. The supernatant was incubated with magnetic bead-conjugated antibodies targeting P300, H3K27ac, H3K4me1, H3K9bhb, and H3K9Ia. Immunocomplexes were washed using LiCl wash buffer (500 mmol/L LiCl, 1% NP-40, 0.5% sodium deoxycholate, 100 mmol/L Tris pH 7.5). DNA extraction for ChIP-qPCR assays involved the addition of Proteinase K and RNase A.

### 2.7. Statistical Analysis

All data were analyzed using GraphPad Prism 9 and presented as mean ± standard deviation (SD). One-way analysis of variance and two-way analysis of variance (ANOVA) were used for statistical analysis to compare the means. Each experimental sample was subjected to technical triplicates. Statistical significance was determined at the levels of * *p* < 0.05, ** *p* < 0.01, and *** *p* < 0.001.

## 3. Results

### 3.1. Cell Growth, Viability, and Differential Gene Enrichment

**Figure 1:**
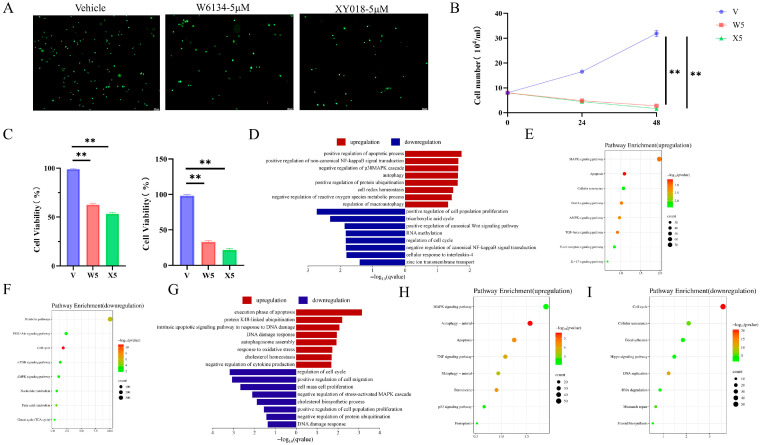
W6134 (5μM) and XY018 (5μM) induce cell death. (**A**) Calcein staining of cells treated with W6134 or XY018 for 48 h (scale bar = 100 μm); (**B**) cell counting after 48 h treatment with W6134 or XY018; (**C**) CCK8 cell viability assays at 24 h and 48 h post-treatment with W6134 or XY018; (**D**) GO analysis showing upregulated pathways enriched in apoptosis and downregulated pathways enriched in cell proliferation and cycle in W6134 cells; (**E**,**F**) KEGG analysis demonstrating upregulated pathways enriched in apoptosis and downregulated pathways enriched in cell cycle in W6134 cells; (**G**) GO analysis showing upregulated pathways enriched in apoptosis and downregulated pathways enriched in cell proliferation and cycle in XY018 cells; (**H**,**I**) KEGG analysis demonstrating upregulated pathways enriched in apoptosis and downregulated pathways enriched in cell cycle in XY018 cells. Figure (**B**) was statistically analyzed using the two-way ANOVA, and figure (**C**) was statistically analyzed using the one-way ANOVA. Data are presented as the means ± SEM, n = 3. Statistical significance is indicated as follows: ** *p* < 0.01.

To assess the potential protective effects, CMT-N7 cells treated with W6134 (5 μM) and XY018 (5 μM) underwent live cell staining, cell counting, and viability assays. Live cell staining revealed that 48-hour treatment with W6134 (5 μM) and XY018 (5 μM) significantly induced a reduction in cell number (Figure 1A). Cell counting and viability data showed significant decreases in cell numbers at 0, 24, and 48 h in W6134 (5 μM) and XY018 (5 μM) groups compared to the control (Figure 1B,C).

A total of 29,993 genes were uncovered from all samples, comprising 14,388 annotated genes (47.97%) and 15,605 unannotated novel genes (52.03%). Among these, 9498 genes (FPKM > 0) were differentially expressed (DE) in W6134 (5 μM) and XY018 (5 μM) groups, which were further subjected to GO functional annotation analysis. In two treatment groups relative to the vehicle group, DE genes were screened using FPKM ratios with thresholds set as FC > 1.5 for upregulation and FC < 0.7 for downregulation. The W6134 5 μM group identified 2495 DEGs, including 791 upregulated genes (*DHX58*, *SLC16A14*, *SLC16A6*) and 1704 downregulated genes (*CCNB1*, *TROAP*, *OIP5*). The XY018 5 μM group identified 1308 DEGs, including 761 upregulated genes *(CYP1A1*, *HECW1*, *RASGEF1B*) and 547 downregulated genes (*LNX1*, *FRMD5*, *AUNIP*).

To identify key transcriptional pathways regulated by W6134 and XY018, Fold Change of FPKM (excluding zero and non-significant values) was calculated, and enrichment analysis was performed using GSEA 4.1.0 and DAVID. As shown in Figure 1D–F, GO and KEGG analyses indicated that upregulated genes in W6134 5 μM-treated cells at 48 h were significantly enriched in apoptosis pathways, while downregulated genes were enriched in cell proliferation and cycle pathways, suggesting that W6134 as an RORγ inhibitor may induce CM-N7 cell death. Similarly, XY018 5 μM treatment at 48 h showed DEGs enriched in apoptosis, proliferation, and cycle pathways (Figure 1G–I), reinforcing RORγ inhibitors’ anticancer effects.

**Figure 2:**
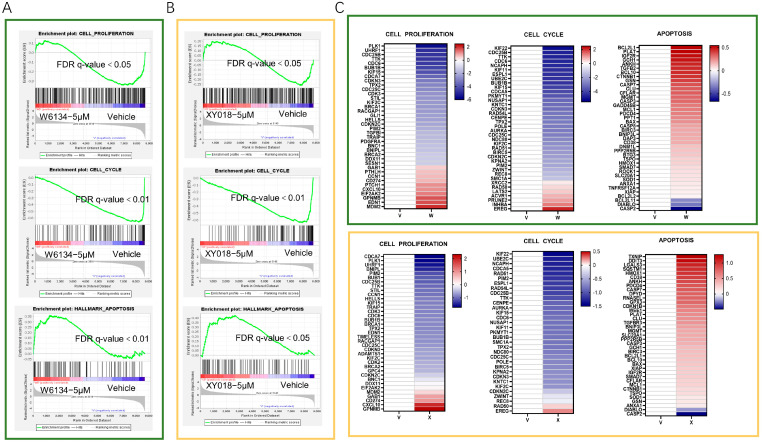
W6134 (5μM) and XY018 (5μM) alter expression of cell death-related genes. (**A**) GSEA plots of cell proliferation, cell cycle, and apoptosis pathways in W6134-treated cells; (**B**) GSEA plots of cell proliferation, cell cycle, and apoptosis pathways in XY018-treated cells; (**C**) heatmap of mRNA expression changes (RNA-seq, log_2_-transformed) in genes related to cell proliferation, cell cycle, and apoptosis pathways after W6134 and XY018 treatments.

GSEA analysis of RNA-seq data further demonstrated that W6134 and XY018 significantly upregulated apoptosis pathways and downregulated cell cycle/proliferation pathways in CMT-N7 cells (Figure 2A,B), with consistent expression trends in apoptosis, cycle, and proliferation genes (Figure 2C).

### 3.2. W6134 and XY018 Regulate Inflammation-Related Pathways

**Figure 3:**
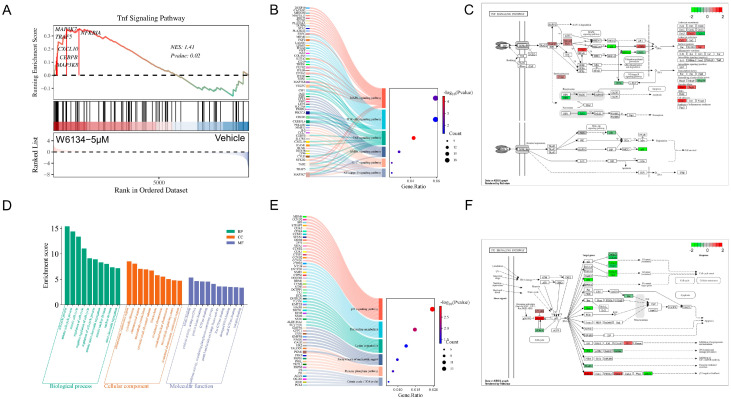
GSEA, KEGG, and GO enrichment analyses of DEGs, showing GSEA enrichment plots, enrichment bubble plots, pathway diagrams, and GO enrichment maps. (**A**) GSEA analysis outlining pathways pertinent to differentially expressed genes in W6134-treated canine cancer cells relative to the control group. (**B**) KEGG enrichment analysis of upregulated DEGs. *X*-axis: enrichment ratio; *Y*-axis: KEGG pathways. Count: bubble size indicates the number of genes annotated to KEGG pathways. *P*: color gradient represents enrichment significance. Left panel: gene names corresponding to each pathway. (**C**) TNF signaling pathway diagram. (**D**) GO enrichment analysis results. (**E**) KEGG enrichment analysis of downregulated DEGs. *X*-axis: enrichment ratio; *Y*-axis: KEGG pathways. Count: bubble size reflects gene number annotated to pathways. *p*-value: color indicates enrichment significance. Left panel: gene names for each pathway. (**F**) p53 signaling pathway diagram.

**Figure 4:**
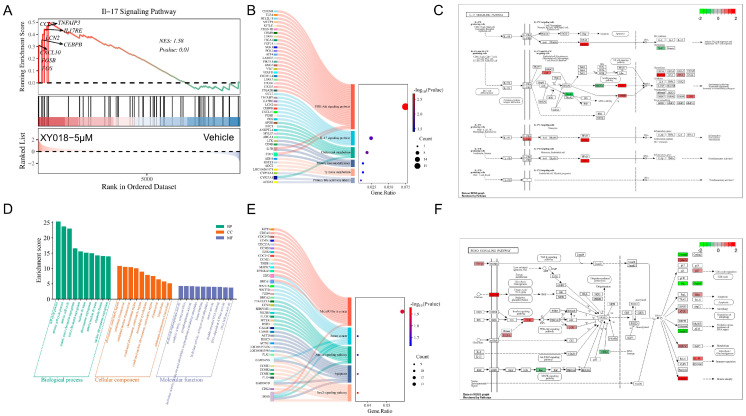
GSEA, KEGG, and GO enrichment analyses of DEGs, showing GSEA enrichment plots, enrichment bubble plots, pathway diagrams, and GO enrichment maps. (**A**) GSEA analysis outlining pathways pertinent to differentially expressed genes in XY018-treated canine cancer cells relative to the control group. (**B**) KEGG enrichment analysis of upregulated DEGs. *X*-axis: enrichment ratio; *Y*-axis: KEGG pathways. Count: Bubble size indicates the number of genes annotated to KEGG pathways. *p*-value: Color gradient represents enrichment significance. Left panel: Gene names corresponding to each pathway. (**C**) IL-17 signaling pathway diagram. (**D**) GO enrichment analysis results. (**E**) KEGG enrichment analysis of downregulated DEGs. *X*-axis: enrichment ratio; *Y*-axis: KEGG pathways. Count: Bubble size reflects the number of genes annotated to pathways. *p*-value: Color indicates enrichment significance. Left panel: Gene names for each pathway. (**F**) FoxO signaling pathway diagram.

RNA-seq results showed that the TNF signaling pathway was highly enriched in the vehicle vs. W6134-5 μM group, with annotated gene names representing core inflammatory genes in the enriched pathway, including *MAP3K8*, *CEBPB*, *CXCL10*, *TRAF5*, *MAP3K7*, and *NFKBIA* (Figure 3A). Among them, *MAP3K7*, highly associated with inflammatory responses, was significantly upregulated (FC = 2.5) after W6134-5 μM treatment (16). In the vehicle vs. XY018-5 μM group, the IL-17 signaling pathway was highly enriched, with core inflammatory genes including *FOS*, *FOSB*, *CXCL10*, *CEBPB*, *LCN2*, *IL17RE*, *TNFAIP3*, and *CCL7* (Figure 4A). Notably, *CXCL10*, strongly linked to inflammation (17), showed an FC of 3.8 and was significantly upregulated after XY018-5 μM treatment.

KEGG enrichment analysis of upregulated DEGs in the W6134-treated group relative to the vehicle revealed significant enrichment in inflammation-related pathways, including the NF-κB signaling pathway, IL-17 signaling pathway, AMPK signaling pathway, TNF signaling pathway, PI3K-Akt signaling pathway, and MAPK signaling pathway. Multiple cross-pathway genes, such as *MAP3K7*, *TRAF5*, and *TAB2*, were identified (Figure 3B). Using LOG_2_^FC^ values as quantitative data, gene expression changes were mapped and visualized on the TNF signaling pathway diagram, enabling systematic observation of core gene expression (Figure 3C). Downregulated DEGs were enriched in pathways including the Citrate cycle (TCA cycle), Pentose phosphate pathway, biosynthesis of nucleotide sugars, Lysine degradation, Pyrimidine metabolism, and p53 signaling pathway (Figure 3E), with related genes mapped to the most significantly enriched p53 signaling pathway diagram (Figure 3F).

GO enrichment analysis (left to right: top 10 enriched pathways in Biological Process, Cellular Component, and Molecular Function) showed DEGs were enriched in 30 pathways, including the cell cycle process, mitotic cell cycle process, mitotic cell cycle, regulation of cell cycle, negative regulation of cell cycle, regulation of cell cycle process, mitotic sister chromatid segregation, mitotic nuclear division, nuclear division, sister chromatid segregation, chromosome centromeric region, cytosolic ribosome, chromosomal region, and microtubule cytoskeleton (Figure 3D).

XY018-treated group’s upregulated DEGs were subjected to KEGG enrichment analysis, primarily concentrated in the PI3K-Akt signaling pathway, cholesterol metabolism, IL-17 signaling pathway, primary bile acid biosynthesis, Tyrosine metabolism, and primary immunodeficiency. Multiple cross-pathway genes, such as *IL7R* and *CD19*, were identified (Figure 4B). By using LOG_2_^FC^ values as quantitative data and mapping/rendering them onto the IL-17 signaling pathway diagram, expression changes of core genes were systematically visualized (Figure 4C). Downregulated DEGs were significantly enriched in KEGG pathways including FoxO signaling pathway, apoptosis, Apelin signaling pathway, breast cancer, and MicroRNAs in cancer (Figure 4E). Related genes were mapped onto the most significantly enriched FoxO signaling pathway diagram (Figure 4F). GO enrichment analysis (left to right: top 10 enriched pathways in Biological Process, Cellular Component, and Molecular Function) showed DEGs were enriched in 30 pathways, including cell cycle process, mitotic cell cycle process, mitotic cell cycle, mitotic sister chromatid segregation, sister chromatid segregation, nuclear division, chromosome segregation, mitotic nuclear division, organelle fission, nuclear chromosome segregation, chromosome centromeric region, condensed chromosome, and condensed nuclear chromosome (Figure 4D).

### 3.3. Histone Modifications Promote the Transcriptional Activation of CXCL10 and MECOM

RORγ, a nuclear receptor, exerts a key role in multiple biological processes including oxidative stress responses and inflammatory reactions [39]. Existing studies have indicated that this nuclear receptor is linked to inflammatory responses. It directly regulates genes related to the oxidative stress response involved in the inflammatory reaction and simultaneously exerts pro-inflammatory effects through the TNF signaling pathway [40]. RORγ may become a key target to regulate the inflammatory response of canine mammary cancer cells.

**Figure 5:**
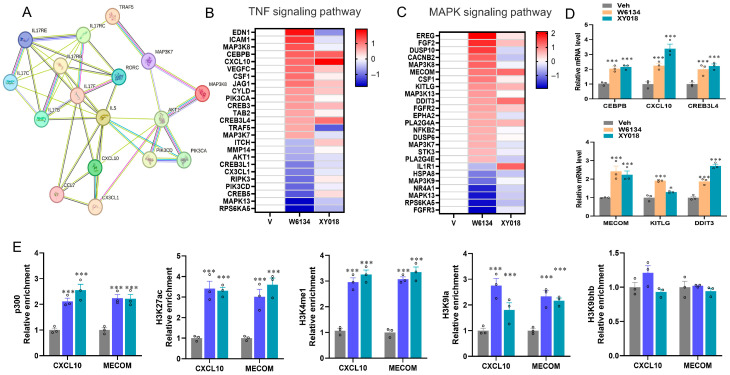
Histone modifications on *CXCL10* and *MECOM* genes. (**A**) STRING-ELIXIR analysis predicted interactions between key differentially expressed genes involved in RORγ transcriptional regulation within the inflammatory response, TNF signaling pathway, and IL-17 signaling pathway. Enrichment *p*-value: <1.0 × 10^−16^. The network view summarizes the network of predicted associations for a particular group of proteins. The network nodes are proteins. The edges represent the predicted functional associations. Red line indicates the presence of fusion evidence. Green line indicates neighborhood evidence. Blue line indicates cooccurrence evidence. Purple line indicates experimental evidence. Yellow line indicates textmining evidence. Light blue line indicates database evidence and Black line indicates coexpression evidence. (**B**,**C**) Heatmaps of differentially expressed genes in TNF/MAPK pathways after W6134 and XY018 treatments. (**D**) Expression levels of core genes in TNF/MAPK pathways after W6134 and XY018 treatments. (**E**) Histone marker measurements by ChIP-qPCR in cells treated with W6134 and XY018. Figures (**D**,**E**) were statistically analyzed using one-way ANOVA. Data are presented as means ± SEM, n = 3. Statistical significance is indicated as follows: * *p* < 0.05, *** *p* < 0.001.

In this study, compared to the vehicle group, most of the differentially expressed genes in canine cancer cells treated with W6134 and XY018 were concentrated and enriched in pathways related to the inflammatory response. Therefore, the key differentially expressed genes in the inflammatory response pathway and the TNF signaling pathway were selected in this study. Additionally, we conducted STRING analysis to predict the associations between these genes and RORC. These results showed that they were highly correlated and connected by 44 edges. *IL-5*, *IL-17RC*, and *IL-17F* interact with RORγ (Figure 5A). These data suggest that RORC may lead to the death of canine mammary cancer cells by promoting the inflammatory response in canine cancer cells.

Research has indicated that RORγ is highly correlated with the inflammatory pathways in breast cancer [41]. We screened the expression values of differentially expressed genes in the TNF signaling pathway and MAPK inflammation-related signaling pathways, performed data normalization (log (treatment/vehicle, 2), log (*v*/*v*, 2)), and drew a heatmap using Graphpad (Figure 5B,C). Among them, pro-inflammatory genes such as CXCL10 and VEGFC were significantly upregulated. The expression levels of core genes CXCL10 and MECOM were detected by q-PCR in cells treated with W6134 and XY018 (Figure 5D).

Given the epigenetic control over primary immunodeficiency and TNF/MAPK signaling pathway regulation, ChIP-qPCR was used to detect histone marker enrichment in *CXCL10* and *MECOM* genes. Histone markers including P300, H3K27ac, H3K4me1, H3K9la, and H3K9bhb antibodies were assayed. Treatment with W6134 and XY018 significantly increased histone markers associated with transcriptional activation (P300, H3K27ac, H3K4me1, H3K9la), but not H3K9bhb (Figure 5E).

## 4. Discussion

Mammary cancer is the most common tumor in female dogs who are not spayed and one of the most frequent malignant neoplasms in dogs. A range of biological, pathological, cultural, and socioeconomic factors—including hormonal status, breed, advanced age, obesity, and diet—contribute to the marked variability in the prevalence of breast tumors [11]. In this study, a comprehensive transcriptomic analysis was conducted to explore the effects of W6134 and XY018 on canine mammary epithelial cells. This analysis revealed that genes and pathways related to apoptosis and inflammation were significantly regulated. The research results highlight the potential significance of W6134 and XY018 in the prevention and treatment of canine mammary cancer and also contribute to a deep-level understanding of their underlying mechanisms of action.

In this study, it was also observed that genes associated with the TNF signaling pathway and the IL-17 signaling pathway were upregulated. According to relevant studies, these pathways exert a key function in biological processes such as inhibiting cancer cell growth [42,43]. The regulatory effects of W6134 and XY018 on these pathways imply that they may inhibit the growth and metastasis of canine mammary cancer cells through certain potential mechanisms, which provides strong support for their application as anticancer agents.

Additionally, in CMT-N7 cells, TNF/MAPK inflammatory pathway-related genes such as *CXCL10* and *MECOM* were significantly upregulated following W6134 and XY018 treatment, exhibiting favorable anti-tumor effects, which aligns with previous findings [25,28]. The upregulation of CXCL10 is particularly significant, as studies have identified that CXCL10 overexpression serves as a marker of favorable prognosis in breast cancer [44]. Meanwhile, the increased expression of CXCL10 may result from the upregulation of upstream anti-tumor immune responses, thereby promoting the infiltration of T cells and NK cells and triggering immune responses [45]. Previous research has shown that MECOM, as a transcription factor, can recruit histone acetyltransferases [46], and may also interact with co-activators to regulate pathways related to the cell cycle and others [47].

Epigenetic regulation plays a critical role in cancer development, and treating breast cancer through epigenetic approaches such as histone modifications represents a potential therapeutic strategy [48]. Histone acetylation is a dynamic and reversible process primarily regulated by histone acetyltransferases and histone deacetylases [49,50]. In contrast to acetylation, histone methylation is a more complex regulatory process, where some markers (H3K9, H3K4) are associated with transcriptional activation and others (H3K27) with transcriptional repression [51]. Many studies have used H3K9bhb chromatin immunoprecipitation (ChIP) techniques to identify BHB-regulated genes under ketogenic diets, BHB therapy, or famine [52,53]. In addition, histone H3K9bhb is linked with starvation-responsive gene regulation and separates some of these genes from those identified by H3K9ac and H3K4me3, indicating a specific role in integrating metabolic and epigenetic regulation in response to starvation [54]. In this study, we detected histone markers in cells treated with W6134 and XY018 using ChIP-qPCR. Compared with the control group, the treatment groups showed increased levels of P300, H3K27ac, H3K4me1, and H3K9la, but not H3K9bhb. Lysine β-hydroxybutyrylation (Kbhb), a newly discovered post-translational modification [55], links ketone metabolism to cell signaling and genome regulation [56].

Notably, the translational relevance of the results of this study is of great importance. Given the certain degree of similarity in mammary physiology and tumor pathology between dogs and humans, the canine model is frequently employed as a substitute model in human breast cancer research [57,58]. Consequently, the findings of this study could carry wider significance for elucidating the roles of W6134 and XY018 in the prevention and treatment of human breast cancer, as well as their potential value as therapeutic drugs.

However, this study also has certain limitations. Since the experiments were carried out in an in vitro environment, the effects of W6134 and XY018 on the entire organism remain unclear. Future research should introduce in vivo models to verify the findings of this study and further explore the long-term effects of W6134 and XY018 in the prevention and treatment of canine mammary cancer. Furthermore, the specific concentrations of W6134 and XY018 utilized in this study might not precisely align with the dosage standards in practical applications, indicating that it is necessary to conduct dose–response studies to determine the optimal therapeutic dose.

In the field of cancer research, animal models with spontaneous tumors are widely regarded as ideal models for studying human cancers [59]. Canine mammary tumors have attracted significant attention due to their high similarity to human breast cancers in terms of tumor size, growth rate, and clinical staging [60]. Ovarian steroids are key risk factors for CMT. Early ovariohysterectomy can effectively prevent the occurrence of CMT, but only a portion of female dogs undergo this surgery. As a result, CMT is often detected at an advanced stage, leading to poor prognosis, and there is a lack of relevant research and treatment options [61]. Therefore, early and accurate diagnosis of CMT, the development of novel treatment strategies, and the promotion of personalized medicine are particularly urgent. In cancer treatment, the exploration of biomarkers and the adoption of personalized treatment have significant advantages [62]. Although the individual differences among cancer patients highlight the necessity of personalized treatment, data on personalized treatment in the field of veterinary oncology are still scarce [63]. Recent preliminary studies based on canine mammary models have indicated that personalized treatment represents an important challenge for future research [64].

Generally, this transcriptomic analysis provides valuable insights into the possible molecular mechanisms by which W6134 and XY018 exert anticancer effects on canine mammary epithelial cells. The regulatory roles of key genes and pathways in the processes of cell inflammation and apoptosis demonstrate the potential of W6134 and XY018 as targeted drugs for the treatment of canine mammary cancer. To further explore these research results in an in vivo environment and translate these insights into practical clinical applications, further research is highly necessary.

## 5. Conclusions

This study investigates the effects of W6134 and XY018 on canine mammary epithelial cells, revealing their significant regulatory roles in cell apoptosis and inflammation-related genes and pathways via histone modifications, which highlights their potential significance in the prevention and treatment of canine mammary cancer and the value of mechanism analysis. The study found that W6134 and XY018 upregulate the expression of key inflammatory genes *CXCL10* and *MECOM*, while genes related to TNF and IL-17 signaling pathways are significantly upregulated, indicating that W6134 and XY018 may exert their anticancer effects by modulating the aforementioned pathways, thereby influencing the growth of cancer cells. The detected elevation in histone acetylation and methylation modifications at specific gene loci indicates that RORγ inhibitors might establish a more conducive transcriptional milieu for genes associated with inflammatory regulation. Given the high similarity between dogs and humans in mammary physiology and tumor pathology, this study is not only of great significance for the prevention and treatment of canine mammary cancer but also provides a broad reference for understanding its role in human breast cancer. Overall, this study provides valuable insights into the molecular mechanisms of anticancer action and preliminarily validates the potential of W6134 and XY018 as targeted drugs for canine mammary cancer. In the future, it will be necessary to carry out further in vivo studies and clinical translation experiments.

## Data Availability

The original data has been uploaded to NCBI PRJNA1223973.

## References

[1] Ishigami-Yuasa M., Kagechika H. (2020). Chemical Screening of Nuclear Receptor Modulators. Int. J. Mol. Sci..

[2] Doan T.B., Graham J.D., Clarke C.L. (2017). Emerging functional roles of nuclear receptors in breast cancer. J. Mol. Endocrinol..

[3] Jin L., Martynowski D., Zheng S., Wada T., Xie W., Li Y. (2010). Structural Basis for Hydroxycholesterols as Natural Ligands of Orphan Nuclear Receptor RORγ. Mol. Endocrinol..

[4] Adam S.Y., Muniyappan M., Huang H., Ennab W., Liu H.Y., Ahmed A.A., Sun M.A., Dessie T., Kim I.H., Hu Y. (2024). Dietary Organic Zinc Supplementation Modifies the Oxidative Genes via RORγ and Epigenetic Regulations in the Ileum of Broiler Chickens Exposed to High-Temperature Stress. Antioxidants.

[5] Huang M., Bolin S., Miller H., Ng H.L. (2020). RORgamma Structural Plasticity and Druggability. Int. J. Mol. Sci..

[6] Cai D., Wang J., Gao B., Li J., Wu F., Zou J.X., Xu J., Jiang Y., Zou H., Huang Z. (2019). RORgamma is a targetable master regulator of cholesterol biosynthesis in a cancer subtype. Nat. Commun..

[7] Zou H., Yang N., Zhang X., Chen H.W. (2022). RORgamma is a context-specific master regulator of cholesterol biosynthesis and an emerging therapeutic target in cancer and autoimmune diseases. Biochem. Pharmacol..

[8] Billon C., Murray M.H., Avdagic A., Burris T.P. (2019). RORgamma regulates the NLRP3 inflammasome. J. Biol. Chem..

[9] Gao M., Guo L., Wang H., Huang J., Han F., Xiang S., Wang J. (2020). Orphan nuclear receptor RORgamma confers doxorubicin resistance in prostate cancer. Cell Biol. Int..

[10] Valdivia G., Alonso-Diez A., Perez-Alenza D., Pena L. (2021). From Conventional to Precision Therapy in Canine Mammary Cancer: A Comprehensive Review. Front. Vet. Sci..

[11] Vazquez E., Lipovka Y., Cervantes-Arias A., Garibay-Escobar A., Haby M.M., Queiroga F.L., Velazquez C. (2023). Canine Mammary Cancer: State of the Art and Future Perspectives. Animals.

[12] Sorenmo K. (2003). Canine mammary gland tumors. Vet. Clin. N. Am. Small Anim. Pract..

[13] Ontsouka E.C., Bertschi J.S., Huang X., Luthi M., Muller S., Albrecht C. (2016). Can widely used cell type markers predict the suitability of immortalized or primary mammary epithelial cell models?. Biol. Res..

[14] Jitpean S., Hagman R., Strom Holst B., Hoglund O.V., Pettersson A., Egenvall A. (2012). Breed variations in the incidence of pyometra and mammary tumours in Swedish dogs. Reprod. Domest. Anim..

[15] Kim H.W., Lim H.Y., Shin J.I., Seung B.J., Ju J.H., Sur J.H. (2016). Breed- and age-related differences in canine mammary tumors. Can. J. Vet. Res..

[16] Enginler S.O., Akis I., Toydemir T.S., Oztabak K., Haktanir D., Gunduz M.C., Kirsan I., Firat I. (2014). Genetic variations of BRCA1 and BRCA2 genes in dogs with mammary tumours. Vet. Res. Commun..

[17] Nieto A., Pena L., Perez-Alenza M.D., Sanchez M.A., Flores J.M., Castano M. (2000). Immunohistologic detection of estrogen receptor alpha in canine mammary tumors: Clinical and pathologic associations and prognostic significance. Vet. Pathol..

[18] Shin J.I., Lim H.Y., Kim H.W., Seung B.J., Ju J.H., Sur J.H. (2016). Analysis of Obesity-Related Factors and their Association with Aromatase Expression in Canine Malignant Mammary Tumours. J. Comp. Pathol..

[19] Nosalova N., Huniadi M., Hornakova L., Valencakova A., Hornak S., Nagoos K., Vozar J., Cizkova D. (2024). Canine Mammary Tumors: Classification, Biomarkers, Traditional and Personalized Therapies. Int. J. Mol. Sci..

[20] Perez Alenza M.D., Pena L., del Castillo N., Nieto A.I. (2000). Factors influencing the incidence and prognosis of canine mammary tumours. J. Small Anim. Pract..

[21] Zhang X., Huang Z., Wang J., Ma Z., Yang J., Corey E., Evans C.P., Yu A.M., Chen H.W. (2021). Targeting Feedforward Loops Formed by Nuclear Receptor RORgamma and Kinase PBK in mCRPC with Hyperactive AR Signaling. Cancers.

[22] Lucchinetti E., Zaugg M. (2020). RNA Sequencing. Anesthesiology.

[23] Liu M., Guo S., Hibbert J.M., Jain V., Singh N., Wilson N.O., Stiles J.K. (2011). CXCL10/IP-10 in infectious diseases pathogenesis and potential therapeutic implications. Cytokine Growth Factor Rev..

[24] Groom J.R., Luster A.D. (2011). CXCR3 ligands: Redundant, collaborative and antagonistic functions. Immunol. Cell Biol..

[25] Karin N. (2020). CXCR3 Ligands in Cancer and Autoimmunity, Chemoattraction of Effector T Cells, and Beyond. Front. Immunol..

[26] Lv J., Meng S., Gu Q., Zheng R., Gao X., Kim J.D., Chen M., Xia B., Zuo Y., Zhu S. (2023). Epigenetic landscape reveals MECOM as an endothelial lineage regulator. Nat. Commun..

[27] Goyama S., Yamamoto G., Shimabe M., Sato T., Ichikawa M., Ogawa S., Chiba S., Kurokawa M. (2008). Evi-1 is a critical regulator for hematopoietic stem cells and transformed leukemic cells. Cell Stem Cell.

[28] Shimomura K., Hattori N., Iida N., Muranaka Y., Sato K., Shiraishi Y., Arai Y., Hama N., Shibata T., Narushima D. (2023). Sleeping Beauty transposon mutagenesis identified genes and pathways involved in inflammation-associated colon tumor development. Nat. Commun..

[29] Lawrence M., Daujat S., Schneider R. (2016). Lateral Thinking: How Histone Modifications Regulate Gene Expression. Trends Genet..

[30] Zaib S., Rana N., Khan I. (2022). Histone Modifications and their Role in Epigenetics of Cancer. Curr. Med. Chem..

[31] Ma L., Li C., Yin H., Huang J., Yu S., Zhao J., Tang Y., Yu M., Lin J., Ding L. (2023). The Mechanism of DNA Methylation and miRNA in Breast Cancer. Int. J. Mol. Sci..

[32] Ma J., Zhang Y., Li J., Dang Y., Hu D. (2025). Regulation of histone H3K27 methylation in inflammation and cancer. Mol. Biomed..

[33] Miziak P., Baran M., Borkiewicz L., Trombik T., Stepulak A. (2024). Acetylation of Histone H3 in Cancer Progression and Prognosis. Int. J. Mol. Sci..

[34] Hou Y., Yuan Y., Li Y., Wang L., Hu J., Liu X. (2023). The role of histone methylation in renal cell cancer: An update. Mol. Biol. Rep..

[35] Gong Z., Li A., Ding J., Li Q., Zhang L., Li Y., Meng Z., Chen F., Huang J., Zhou D. (2021). OTUD7B Deubiquitinates LSD1 to Govern Its Binding Partner Specificity, Homeostasis, and Breast Cancer Metastasis. Adv. Sci..

[36] Marinova Z., Leng Y., Leeds P., Chuang D.M. (2011). Histone deacetylase inhibition alters histone methylation associated with heat shock protein 70 promoter modifications in astrocytes and neurons. Neuropharmacology.

[37] Hong J., Adam S.Y., Wang S., Huang H., Kim I.H., Ahmed A.A., Liu H.Y., Cai D. (2025). Melatonin Modulates ZAP70 and CD40 Transcripts via Histone Modifications in Canine Ileum Epithelial Cells. Vet. Sci..

[38] Song X.H., Gao S.S., Hu S.H., Fang T., Xu X.L., Lv X., Gao X.G., Lin M.J., Peng L., Li M. (2024). Establishment and Characterization of a New Canine Mammary Cancer Cell Line CMT-N7: Implications for Comparative Oncology and Therapeutic Development. Pak. Vet. J..

[39] Alexander M., Ang Q.Y., Nayak R.R., Bustion A.E., Sandy M., Zhang B., Upadhyay V., Pollard K.S., Lynch S.V., Turnbaugh P.J. (2022). Human gut bacterial metabolism drives Th17 activation and colitis. Cell Host Microbe.

[40] Franken A., Van Mol P., Vanmassenhove S., Donders E., Schepers R., Van Brussel T., Dooms C., Yserbyt J., De Crem N., Testelmans D. (2022). Single-cell transcriptomics identifies pathogenic T-helper 17.1 cells and pro-inflammatory monocytes in immune checkpoint inhibitor-related pneumonitis. J. Immunother. Cancer.

[41] Berkel C., Cacan E. (2024). A majority of circadian clock genes are expressed in estrogen receptor and progesterone receptor status-dependent manner in breast cancer. J. Biosci..

[42] Manohar S.M. (2024). At the Crossroads of TNF alpha Signaling and Cancer. Curr. Mol. Pharmacol..

[43] Wang C., Kong L., Kim S., Lee S., Oh S., Jo S., Jang I., Kim T.D. (2022). The Role of IL-7 and IL-7R in Cancer Pathophysiology and Immunotherapy. Int. J. Mol. Sci..

[44] Chuan T., Li T., Yi C. (2020). Identification of CXCR4 and CXCL10 as Potential Predictive Biomarkers in Triple Negative Breast Cancer (TNBC). Med. Sci. Monit..

[45] Ka N.L., Park M.K., Kim S.S., Jeon Y., Hwang S., Kim S.M., Lim G.Y., Lee H., Lee M.O. (2023). NR1D1 Stimulates Antitumor Immune Responses in Breast Cancer by Activating cGAS-STING Signaling. Cancer Res..

[46] Xu X., Woo C.H., Steere R.R., Lee B.C., Huang Y., Wu J., Pang J., Lim J.H., Xu H., Zhang W. (2012). EVI1 acts as an inducible negative-feedback regulator of NF-kappaB by inhibiting p65 acetylation. J. Immunol..

[47] Yatsula B., Lin S., Read A.J., Poholek A., Yates K., Yue D., Hui P., Perkins A.S. (2005). Identification of binding sites of EVI1 in mammalian cells. J. Biol. Chem..

[48] Szczepanek J., Skorupa M., Jarkiewicz-Tretyn J., Cybulski C., Tretyn A. (2023). Harnessing Epigenetics for Breast Cancer Therapy: The Role of DNA Methylation, Histone Modifications, and MicroRNA. Int. J. Mol. Sci..

[49] Ropero S., Esteller M. (2007). The role of histone deacetylases (HDACs) in human cancer. Mol. Oncol..

[50] Roy S.S., Gonugunta V.K., Bandyopadhyay A., Rao M.K., Goodall G.J., Sun L.Z., Tekmal R.R., Vadlamudi R.K. (2014). Significance of PELP1/HDAC2/miR-200 regulatory network in EMT and metastasis of breast cancer. Oncogene.

[51] Jenuwein T., Allis C.D. (2001). Translating the histone code. Science.

[52] Zheng Y., Sun W., Shan C., Li B., Liu J., Xing H., Xu Q., Cui B., Zhu W., Chen J. (2022). β-hydroxybutyrate inhibits ferroptosis-mediated pancreatic damage in acute liver failure through the increase of H3K9bhb. Cell Rep..

[53] Terranova C.J., Stemler K.M., Barrodia P., Jeter-Jones S.L., Ge Z., de la Cruz Bonilla M., Raman A., Cheng C.W., Allton K.L., Arslan E. (2021). Reprogramming of H3K9bhb at regulatory elements is a key feature of fasting in the small intestine. Cell Rep..

[54] Xie Z., Zhang D., Chung D., Tang Z., Huang H., Dai L., Qi S., Li J., Colak G., Chen Y. (2016). Metabolic Regulation of Gene Expression by Histone Lysine β-Hydroxybutyrylation. Mol. Cell.

[55] Tsusaka T., Oses-Prieto J.A., Lee C., DeFelice B.C., Burlingame A.L., Goldberg E.L. (2023). Non-specific recognition of histone modifications by H3K9bhb antibody. iScience.

[56] Garcia-Velazquez L., Massieu L. (2023). The proteomic effects of ketone bodies: Implications for proteostasis and brain proteinopathies. Front. Mol. Neurosci..

[57] Raposo T.P., Arias-Pulido H., Chaher N., Fiering S.N., Argyle D.J., Prada J., Pires I., Queiroga F.L. (2017). Comparative aspects of canine and human inflammatory breast cancer. Semin. Oncol..

[58] Gherman L.M., Chiroi P., Nutu A., Bica C., Berindan-Neagoe I. (2024). Profiling canine mammary tumors: A potential model for studying human breast cancer. Vet. J..

[59] Oliveira-Lopes A.F., Gotze M.M., Lopes-Neto B.E., Guerreiro D.D., Bustamante-Filho I.C., Moura A.A. (2024). Molecular and Pathobiology of Canine Mammary Tumour: Defining a Translational Model for Human Breast Cancer. Vet. Comp. Oncol..

[60] Abadie J., Nguyen F., Loussouarn D., Pena L., Gama A., Rieder N., Belousov A., Bemelmans I., Jaillardon L., Ibisch C. (2018). Canine invasive mammary carcinomas as models of human breast cancer. Part 2: Immunophenotypes and prognostic significance. Breast Cancer Res. Treat..

[61] Zhang L., Xie Y., Liang X., Yin L., He C., Yin Z., Yue G., Zou Y., Li L., Song X. (2023). Synthesis of structurally diverse derivatives of aconitine-type diterpenoid alkaloids and their anti-proliferative effects on canine breast cancer cells. Bioorg. Chem..

[62] Li D., English H., Hong J., Liang T., Merlino G., Day C.P., Ho M. (2022). A novel PD-L1-targeted shark V(NAR) single-domain-based CAR-T cell strategy for treating breast cancer and liver cancer. Mol. Ther. Oncolytics.

[63] Ke C.H., Lin C.N., Lin C.S. (2024). Hormone, Targeted, and Combinational Therapies for Breast Cancers: From Humans to Dogs. Int. J. Mol. Sci..

[64] Michishita M., Ochiai K., Nakahira R., Azakami D., Machida Y., Nagashima T., Nakagawa T., Ishiwata T. (2023). mTOR pathway as a potential therapeutic target for cancer stem cells in canine mammary carcinoma. Front. Oncol..

